# Cancer related knowledge, attitude, and practice among community health care providers and health assistants in rural Bangladesh

**DOI:** 10.1186/s12913-021-06202-z

**Published:** 2021-03-02

**Authors:** Nazirum Mubin, Redwan Bin Abdul Baten, Sayeeda Jahan, Fatema Tuz Zohora, Naim Mahmud Chowdhury, Golam Mohiuddin Faruque

**Affiliations:** 1 Department of Radiotherapy, Dhaka Medical College Hospital, Dhaka, Bangladesh; 2 Department of Health Management and Policy, University of Iowa, Iowa City, USA; 3 Centre for Policy Dialogue (CPD), Dhaka, Bangladesh; 4 Ministry of Health and Family Welfare, Government of Bangladesh, Dhaka, Bangladesh; 5 Department of Oral and Maxillofacial Surgery, Chhattogram International Dental College, Chittagong, Bangladesh; 6 Bangladesh Cancer Society, Dhaka, Bangladesh

**Keywords:** Healthcare workforce, Community health care provider, Health assistant, Cancer, Knowledge, Attitude, Practice, KAP, Rural health, Community clinics

## Abstract

**Background:**

Cancer remains one of the primary causes of death in Bangladesh. The success of cancer control in rural areas depends on the ability of the health care system and workforce to identify and manage cases properly at early stages. Community Health Workers (CHW) can play a vital role in this process. The present study aims to assess cancer related Knowledge, Attitude, and Practice (KAP) among 2 categories of CHWs - Community Health Care Providers (CHCP) and Health Assistants (HA) in rural Bangladesh.

**Methods:**

A descriptive cross-sectional study was conducted using a self-administered questionnaire from July 2019 to June 2020. Multi-stage sampling technique was used to determine the sample. One Upazilla Health Complex (UHC) from each of the eight administrative divisions of Bangladesh were randomly chosen as study sites, from which 325 CHCPs and HAs were in the final sample. Multivariate logistic regression models were developed to determine the association between KAP scores and demographic variables.

**Results:**

Our study shows that a modest number of respondents scored above average in the knowledge (54.15%), attitude (58.15%), and practice (65.54%) sections. Majority CHCPs (90.91%) and HAs (96.06%) did not receive govt. training on cancer. Only 20.71% HAs and 25.2% CHCPs knew about the availability of cancer treatment options in Bangladesh. Uncertainty about the availability of relevant treatments or vaccinations at public facilities was also high. Having cancer in the family, income, duration of employment and workplace locations were important predictors of cancer related KAP scores.

**Conclusion:**

Healthcare workforce’s knowledge gap and unfavorable attitude towards cancer may result in poor delivery of care at the rural level. For many people in rural areas, CHCPs and HAs are the first point of contact with the healthcare system and thus effective cancer control strategies must consider them as key stakeholders. Targeted training programs must be adopted to address the cancer related KAP gaps among CHCPs and HAs.

**Supplementary Information:**

The online version contains supplementary material available at 10.1186/s12913-021-06202-z.

## Background

Cancer is one of the leading causes of death in Bangladesh. Cancer accounts for 10% of total deaths in Bangladesh [1] and may reach 13% by 2030 [2]. Every year, 200,000 patients are newly diagnosed with cancer in Bangladesh [3]. The five most frequent cancers occurring in Bangladesh are of - breast (12.10%), esophagus (11.30%), cervix uteri (9.70%), lung (8.80%), lip and oral cavity (8.70%) [4].

Prevention of cancer is very important to lower national mortality rates and improve lives of the people [1, 5]. Cancer is a chronic disease which is expensive to treat. The burden of cancer is thus disproportionately higher for indigent people living in rural areas [6, 7]. But cancer related services are scarce at the rural levels of our healthcare system, where there is a dearth of specialized doctors and healthcare providers [8, 9]. One study found that in rural communities where the number of doctors declined, the number of alternative providers increased [10]. Of these alternative providers, Community Health Workers (CHWs) have been used in many settings as a way of filling gaps in service provision where more skilled personnel are not available, particularly in rural Bangladesh [11]. From the many categories of alternative providers serving at the rural levels of Bangladesh, we are considering Community Health Care Providers (CHCP) and Health Assistants (HA) as CHWs in our study.

Bangladesh has several administrative units such as divisions, districts, upazillas, union parishads and villages. Each upazilla has its own Upazilla Health Complex (UHC) and the Community Clinics (CC) usually serve at the village level. All CCs operate under its corresponding UHC. In 2009, government took the initiative for revitalizing the rural healthcare system of Bangladesh by focusing on Community Clinics (CCs). Under this project, closed CCs were made functional, all necessary logistics including medicine were provided and capacity-building initiatives were launched. Along with the existing HAs, a new professional category of service provider was created, called CHCPs. It is worth noting that CHCPs are employed under this project, while the HAs are directly employed by the govt. There are a few Doctors posted at each UHC, where they provide medical care in both indoor and outdoor settings. The Doctors are supposed to visit the CCs periodically to provide medical care. Unfortunately, due to resource constraints and manpower shortages, the CHCPs and HAs provide most of the care at the CC level. The job description of CHCPs include providing essential medical, nutritional, and family planning services at the CCs. They also need to keep an eye out for patients of complicated or infectious diseases such as tuberculosis, leprosy or kala azar [12]. CHCPs work to assist the HAs and HAs are primarily responsible for operating the CCs, while providing essential care and maintaining a digital database [13]. In addition to serving at the CCs, both CHCPs and HAs are instructed to make house visits, keep lists of complicated patients, pregnancies, administer vaccines at homes etc. Therefore, they have a better opportunity to identify possible cancer patients at the rural level.

Due to the efforts of CHWs, Bangladesh has achieved the health-related goals of the Millennium Development Goals (MDG) by 2015 [9, 14]. The Millennium Development Goals (MDGs) were established by the United Nations (UN) in 2000. There were 8 goals to be achieved by the year 2015. These included important health related goals such as reducing child mortality, improving maternal health, combating HIV/AIDS, malaria, and other diseases, eradicating extreme poverty and hunger etc. For a developing nation like Bangladesh, achieving these goals were very important as it was lagging in several of these indicators. To meet the goals, the govt. focused on rural CHWs such as CHCPs and HAs. CHCPs and HAs worked very hard and were instrumental in achieving the MDG goals within the deadline. While working to achieve the MDG goals, the CHWs developed a grassroot network, database and gained experience by making extensive home visits. All these resources can be retooled and repurposed in developing the cancer tackling strategies in Bangladesh.

As of November 2018, 13,779 CCs (one for every 6000 people) were operational, which were staffed with 13,507 CHCPs and 15,420 HAs. From April 2009 to November 2018, there were 739.70 million visits by rural people to CCs all over Bangladesh and 13.229 million emergency and complicated cases were referred to higher facilities for better management [15]. This referral system is very important to ensure complicated cases are treated by the proper specialists [16]. For most rural people, CHWs thus act as the first point of contact with the govt. healthcare system.

As the frontline healthcare workforce [17], CHWs can play a vital role in reducing the burden of cancer in rural areas. A national strategy on cancer is necessary to establish a proper referral chain between rural and urban components of the healthcare system [18]. Previous studies have highlighted the importance of integrating cancer prevention strategies at all levels of the healthcare delivery system [19].

Lack of knowledge can affect cancer related practices and attitudes among CHCPs and HAs. Currently there is a dearth of information regarding CHWs' KAP levels, which is essential for designing a comprehensive cancer prevention and control strategy [19]. The aim of this study is to assess cancer related KAP among CHCPs and HAs; to understand the factors associated with each domain; to identify gaps; and provide appropriate policy suggestions.

## Methods

### Study design and setting

We conducted a cross-sectional study to assess cancer related KAP among CHCPs and HAs. Data was collected between July 2019 to June 2020. At the time of study, there were 8 administrative divisions, 64 districts and 492 upazillas in Bangladesh [4]. We utilized a multi-stage sampling technique to determine the sample at the upazilla level [20].

### Study sample and sampling technique

**Fig. 1:**
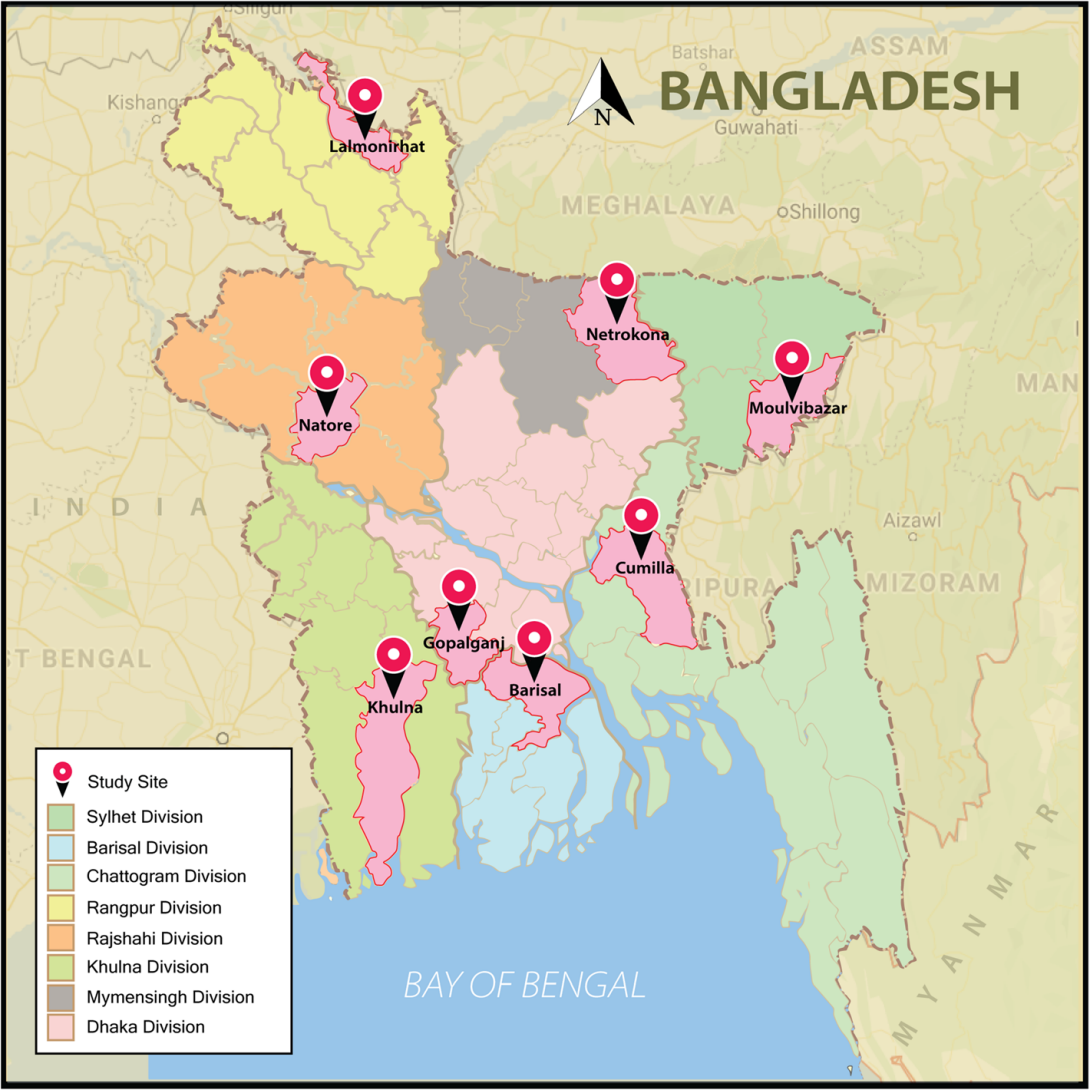
Locations of the study sites at the 8 divisions of Bangladesh

In the first stage, one UHC from each of the eight divisions was randomly selected as our study sites [20]. The random selection was done through Microsoft Excel from a complete list of UHCs in each division. The UHCs were de-identified to protect the identities of the respondents. The study sites were in the following districts and divisions (Fig. 1) – Gopalganj (Dhaka); Cumilla (Chattogram); Natore (Rajshahi); Moulvibazar (Sylhet); Khulna (Khulna); Barisal (Barisal); Netrokona (Mymensingh); and Lalmonirhat (Rangpur).

In the second stage, CHCPs and HAs working under each UHCs were approached to participate in this study. From each UHC, a list of all working CHCPs and HAs was obtained, which served as our sampling frame. Convenience sampling technique was employed at this stage to recruit participants for our study [21]. At every UHC in Bangladesh, a monthly meeting is held where all staff including CHCPs and HAs participate. At each study site, one of these monthly meetings were conveniently chosen to administer the survey among participating CHCPs and HAs.

Due to the descriptive nature of our study and unavailability of previous information about the topic, we did not calculate sample size using any formula. We aimed to recruit at least 50% of all CHCPs and HAs working at the study sites and expected that this would allow us to improve our understanding of cancer related KAP among CHWs in Bangladesh [18]. The total number of HAs was 303 in the 8 selected UHCs, of which 198 (65.35%) participated in the study. For CHCPs, the total number was 239 in the 8 UHCs and among them, 127 (53.14%) participated in our survey. The overall response rate was 59.96%, with a sample size of 325.

### Study instrument

A self-administered KAP questionnaire comprising of 4 sections was used for data collection (Additional file 1). An extensive literature review revealed a dearth of published articles in the field of study. The authors took guidance from the published literature in forming the questionnaire. But due to lack of existing studies, some items of the questionnaire were constructed from clinical knowledge and practical experience as well. We relied on World Health Organization (WHO) recommendations [22] as well to form the questionnaire. In addition to demographic data, 19 items were used to assess knowledge, 15 items focused on attitude and 6 items addressed cancer related practices. The respondents were asked open and close ended questions. The close ended questions had binary and continuous answer options.

The questionnaire was initially prepared in English and then translated to Bengali for easy administration [21]. The Bengali version of the questionnaire was pre-tested [19] among a small group (*n* = 10) of CHCPs and HAs at a UHC located in Gopalganj, Dhaka. The pre-testing was done among a small sample due to resource constraints and budgetary reasons. Most of the feedback were related to linguistic issues or inability to understand certain technical terms. The authors modified the language of the questionnaire items to make it easier for the respondents.

### Statistical analysis

To understand the demographic characteristics of the respondents, descriptive statistics were analyzed for each professional category of CHCPs and HAs. Chi-square tests (Mantel-Haenszel for ordinal variables and Likelihood ratio for nominal variables) were performed to see the association between professional categories and demographic variables.

The outcomes of interest were - total individual score in each section of knowledge, attitude, and practice (dependent variables). In the models we controlled for - age, sex, duration of employment, professional category, religion, cancer in the family, marital status, education, smoking status, income, and job location (independent variables).

To determine the score for each category, a pre-determined set of correct answers were used. A score of one was assigned to each correct option (binary and categorical), which resulted in a total score of 74 for knowledge, 15 for attitude and 6 for practice. The aggregate score for every respondent was then calculated, followed by the mean for each for the 3 sections. The mean of each category was used as a cut-off point to determine if the respondents did ‘good’ or ‘bad’ in each section [23]. To assess the probability of doing good or bad in each section, separate multivariate logistic regression models were developed [18]. The results of all logistic regression analysis were reported as odds ratios (OR) with a 95% confidence interval (95% CI). Final analyses were completed using Stata 16.1 software (StataCorp) for windows.

### Ethical considerations

The study was performed in accordance with the Declaration of Helsinki and the study protocol passed through the institutional review process at the Bangladesh Cancer Society for ethical approval. Official letters of permission were obtained from respective administrative officials of the UHCs. The participants were informed about the objectives of the study and confidentiality issues before administration of the survey. All study participants gave informed consent. Anonymity of the participants and their work locations was ensured throughout the study by de-identification.

### Role of the funding source

The study was funded by the ‘Prof. SF Haque – Dr. SH Grant’ provided by Bangladesh Cancer Society. The society had no role in study design, data collection, analysis, or preparation of manuscript for the study.

## Results

Table 1 report the demographic characteristics of the study sample stratified by professional category. Results for cancer related Knowledge (Table 2), Attitude (Table 3), and Practice (Table 4) are also reported by professional category. Tables 5 and 6 report descriptive and multivariate logistic regression analysis of respondents’ KAP scores.

**Table 1:** Demographic characteristics of HAs and CHCPs

Characteristics	CHCPs^**%**^	HAs^**%**^	***p***-value
**Age**			
*21–30*	55 (43.31)	26 (13.13)	**0.000**^**$**^
*31–40*	69 (54.33)	94 (47.47)	
*41–50*	2 (1.57)	43 (21.72)	
*51–60*	1 (0.79)	35 (17.68)	
**Sex**			
*Male*	61 (48.03)	102 (51.52)	0.540^&^
*Female*	66 (51.97)	96 (48.48)	
**Education**			
*Secondary School Certificate (SSC)*	2 (1.57)	19 (9.60)	**0.000**^**$**^
*Higher Secondary Certificate (HSC)*	28 (22.05)	69 (34.85)	
*Diploma*	3 (2.36)	9 (4.55)	
*Honors*	22 (17.32)	49 (24.75)	
*Masters*	72 (56.69)	52 (26.26)	
**Marital Status**			
*Single*	16 (12.60)	18 (9.09)	0.318^&^
*Married*	111 (87.40)	180 (90.91)	
**Duration of employment**			
*< 10 years*	114 (89.76)	80 (40.40)	**0.000**^**$**^
*11–20 years*	12 (9.45)	51 (25.76)	
*> 20 years*	1 (0.79)	67 (33.84)	
**Religion**			
*Islam*	96 (75.59)	132 (66.67)	**0.084**^**&**^
*Others*	31 (24.41)	66 (33.33)	
**Smoking**			
*No*	116 (91.34)	177 (89.39)	0.563^&^
*Yes*	11 (8.66)	21 (10.61)	
**Monthly Income**			
*< 15,000*	11 (8.66)	19 (9.60)	**0.000**^**$**^
*15,001–20,000*	109 (85.83)	87 (43.94)	
*20,001–25,000*	2 (1.57)	45 (22.73)	
*> 25,000*	5 (3.94)	47 (23.74)	
**Cancer in the family**			
*No*	122 (96.06)	182 (91.92)	0.126^&^
*Yes*	5 (3.94)	16 (8.08)	
**Workplace Location**			
*Moulvibazar, Sylhet*	11 (8.66)	40 (20.20)	**0.000**^**&**^
*Barisal, Barisal*	18 (14.17)	17 (8.59)	
*Cumilla, Chattogram*	37 (29.13)	32 (16.16)	
*Lalmonirhat, Rangpur*	21 (16.54)	27 (13.64)	
*Natore, Rajshahi*	15 (11.81)	12 (6.06)	
*Khulna, Khulna*	1 (0.79)	29 (14.65)	
*Netrokona, Mymensingh*	15 (11.81)	18 (9.09)	
*Gopalganj, Dhaka*	9 (7.09)	23 (11.62)	

^%^ N (%); ^&^ Likelihood ratio Chi-square, ^$^ Extended Mantel-Haenszel (Cochran-Mantel-Haenszel) Stratified Test of association

**Table 2:** Cancer related knowledge of CHCPs and HAs

Knowledge Items	Correct Answers	CHCP^**%**^	HA^**%**^	***p-***value
*Who can be affected by Cancer?*	Anyone	188 (94.95)	123 (96.85)	0.118
*Is Cancer contagious?*	No	175 (88.38)	118 (92.91)	0.405
*Is Cancer a hereditary disease?*	A few types	78 (39.39)	45 (35.43)	0.183
*What is/are the risk factor(s) for Cancer?* ^*a*^	13 categories			
*Can Cancer be prevented?*	Yes	128 (64.65)	87 (68.50)	0.694
*How can Cancer be prevented?* ^*a*^	7 categories			
*What is/are the warning sign(s) of Cancer?* ^*a*^	15categories			
*What is/are the treatment option(s) for Cancer?* ^*a*^	11 categories			
*Can Cancer be completely cured?*	Yes	68 (34.34)	34 (26.77)	0.322
*Can Cancer be prevented through Vaccination?*	Some are preventable	121 (61.11)	76 (59.84)	0.431
*What types of Cancer are preventable through Vaccination?* ^*a*^	5 categories			
*What are the consequences of incomplete treatment for a Cancer patient?*	Patient will deteriorate	171 (86.36)	111 (87.40)	0.326
*Are all treatment options for Cancer available in Bangladesh?*	Yes	41 (20.71)	32 (25.20)	0.616
*What is the diagnostic test for Cancer?* ^*a*^	3 categories			
*Is there any Cancer Screening Program in govt. hospitals which is free of cost?*	Yes	101 (51.01)	62 (48.82)	0.901
*What types of Cancer are screened as part of this program?* ^*a*^	4 categories			
*Do you have enough training to treat Cancer?*	No	194 (97.98)	125 (98.43)	0.771
*Have you received any govt. training on Cancer?*	No	180 (90.91)	122 (96.06)	0.077
*Where can you treat Cancer?* ^*a*^	4 categories			

^%^ N (%); ^a^ Details of the categories can be found in the attached Additional file 1

**Table 3:** Cancer related attitude of CHCPs and HAs

Attitude Items	Correct Answers	CHCP^**%**^	HA^**%**^	***p-***value
*Do you feel sympathy towards Cancer affected patients?*	Yes	188 (94.95)	115 (90.55)	0.304
*What type of Cancer patients do you feel more sympathetic to?*	Do not differentiate	163 (82.32)	114 (89.76)	0.216
*Are you afraid of Cancer patients?*	No	164 (82.83)	106 (83.46)	0.987
*Do you feel hesitant to speak with Cancer patients?*	No	180 (90.91)	118 (92.91)	0.595
*Do you think that Cancer patients are socially marginalized?*	No	177 (89.39)	110 (86.61)	0.736
*Do you think that Cancer patients are avoided by their friends?*	No	140 (70.71)	93 (73.23)	**0.055**
*Do you think Cancer patients face administrative discrimination in terms of receiving govt. benefits?*	No	118 (59.60)	87 (68.50)	**0.048**
*Do you think a patient is responsible for his own disease/fate?*	No	122 (61.62)	74 (58.27)	0.072
*If you realize a patient has Cancer, will you disclose that information to others?*	No	103 (52.02)	68 (53.54)	0.899
*If you realize a patient is receiving treatment for Cancer, will you disclose that information to others?*	No	79 (39.90)	51 (40.16)	0.999
*Do you think Cancer patients should think less of themselves due to their condition?*	No	56 (28.28)	43 (33.86)	0.562
*Do you think Cancer patients should be ashamed of themselves due to their condition?*	No	183 (92.42)	120 (94.49)	0.760
*Would you be ashamed of yourself if diagnosed with Cancer?*	No	154 (77.78)	109 (85.83)	0.197
*How serious of a disease is Cancer?*	Very	168 (84.85)	98 (77.17)	0.196
*In your opinion, how prevalent is Cancer in Bangladesh?*	High	90 (45.45)	43 (33.86)	0.088

^%^ N (%)

**Table 4:** Cancer related practice of CHCPs and HAs

Practice Items	Correct Answers	CHCP^**%**^	HA^**%**^	***p***-value
*If you realize a patient has cancer, will you try to treat by yourself?*	No	189 (95.45)	125 (98.43)	0.148
*If you realize a patient has Cancer, do you refer him/her to the UHC or any other specialist physician?*	Yes	183 (92.42)	121 (95.28)	0.308
*If referred, does a patient actually go to the UHC?*	Yes	147 (74.24)	102 (80.31)	0.207
*If you realize a patient has Cancer, do you advise the patient to take Homeopathic or Ayurvedic treatment?*	No	196 (98.99)	121 (95.28)	**0.035**
*Do you educate people on Cancer during fieldwork or activities at CC?*	Yes	186 (93.94)	119 (93.70)	0.930
*Do you follow-up on a Cancer patient once identified during fieldwork?*	Yes	169 (85.35)	107 (84.25)	0.787

^%^ N (%)

**Table 5:** Knowledge, Attitude, and Practice scores of CHCPs and HAs

	Knowledge Score	Attitude Score	Practice Score
**Total score**	74	15	6
**Median Score**	48	10	6
**Average Score** ^**a**^	46.77 (46.06, 47.49)	9.67 (9.44, 9.91)	5.43 (5.33, 5.53)
**Above Average**^**%**^	176 (54.15)	189 (58.15)	213 (65.54)
**Below Average**^**%**^	149 (45.85)	136 (41.85)	112 (34.46)
***N***	**325**	**325**	**325**

^%^ N (%), ^a^ 95% Confidence Interval in parenthesis

**Table 6:** Multivariate Logistic Regression Models for Above/Below Average of Knowledge, Attitude and Practice Scores

	Above Average Knowledge Score^**$**^	Above Average Attitude Score^**$**^	Above Average Practice Score^**$**^
**CHCP** (Ref.: HA)	1.13 (0.61, 2.08)	0.75 (0.40, 1.40)	1.21 (0.59, 2.48)
**Age** (Ref.: 21–30)			
*31–40*	1.07 (0.57, 2.02)	1.23 (0.64, 2.37)	0.80 (0.40, 1.61)
*41–50*	0.91 (0.24, 3.46)	1.03 (0.31, 3.39)	0.93 (0.25, 3.56)
*51–60*	1.41 (0.30, 6.72)	2.09 (0.52, 8.36)	1.10 (0.22, 5.49)
**Sex** (Ref.: Male)	1.15 (0.69, 1.90)	1.08 (0.65, 1.80)	0.98 (0.57, 1.67)
**Education (Ref.: HSC)**			
*SSC*	1.01 (0.35, 2.95)	1.02 (0.38, 2.76)	1.10 (0.37, 3.26)
*Diploma*	1.53 (0.35, 6.74)	0.88 (0.24, 3.23)	1.19 (0.27, 5.16)
*Honors*	0.98 (0.48, 2.03)	1.18 (0.57, 2.44)	0.59 (0.25, 1.40)
*Masters*	1.19 (0.65, 2.17)	0.99 (0.52, 1.89)	0.91 (0.45, 1.87)
**Marital Status** (Ref.: Single)	3.30*** (1.38, 7.87)	0.68 (0.29, 1.59)	0.92 (0.37, 2.29)
**Duration of employment** (Ref.: < 10 years)			
*11–20 years*	0.94 (0.42, 2.12)	0.40** (0.18, 0.88)	1.12 (0.45, 2.81)
*> 20 years*	0.91 (0.23, 3.58)	2.34 (0.67, 8.15)	1.02 (0.22, 4.65)
**Religion** (Ref.: Others)	0.75 (0.42, 1.35)	1.64* (0.92, 2.91)	1.61 (0.84, 3.10)
**Smoking** (Ref.: No)	0.68 (0.31, 1.50)	0.67 (0.30, 1.48)	2.45* (0.95, 6.33)
**Monthly Income** (Ref.: < 15,000)			
*15,001–20,000*	0.53 (0.20, 1.42)	0.99 (0.37, 2.64)	0.34 (0.09, 1.26)
*20,001–25,000*	0.73 (0.23, 2.34)	0.83 (0.26, 2.58)	0.65 (0.17, 2.49)
*> 25,000*	0.59 (0.18, 1.94)	0.29* (0.08, 1.13)	0.17** (0.04, 0.81)
**Cancer in the family** (Ref.: No)	2.48* (0.87, 7.05)	1.56 (0.52, 4.72)	3.80* (0.96, 15.03)
**Workplace Location** (Ref.: Gopalganj, Dhaka)			
*Moulvibazar, Sylhet*	5.72*** (1.90, 17.30)	3.11** (1.05, 9.19)	7.24*** (2.22, 23.62)
*Barisal, Barisal*	4.50*** (1.51, 13.46)	2.13 (0.72, 6.33)	1.39 (0.45, 4.23)
*Cumilla, Chattogram*	1.66 (0.59, 4.70)	1.29 (0.43, 3.86)	0.54 (0.17, 1.73)
*Lalmonirhat, Rangpur*	1.46 (0.54, 3.96)	1.26 (0.44, 3.58)	3.99** (1.30, 12.30)
*Natore, Rajshahi*	1.13 (0.34, 3.71)	2.93 (0.77, 11.08)	3.53* (0.96, 13.00)
*Khulna, Khulna*	2.34 (0.74, 7.41)	0.66 (0.21, 2.11)	0.63 (0.20, 1.95)
*Netrokona, Mymensingh*	0.87 (0.28, 2.67)	1.69 (0.53, 5.35)	1.84 (0.54, 6.28)

^$^ Odds Ratios; 95% Confidence Interval in parentheses; ^*^ *p* < 0.10, ^**^ *p* < 0.05, ^***^ *p* < 0.01

### Socio-demographic characteristics

Table 1 shows that that there was statistically significant association between professional category and age, education, duration of employment, religion, monthly income, and workplace location. We found that a majority of CHCPs were younger in age whereas the age of HAs was more evenly spread over the 4 age categories. Most of the CHCPs had a shorter duration of employment than the HAs. We saw that both CHCPs and HAs had an even mix of males and females. Most HAs and CHCPs were married (HA - 90.91%; CHCP - 87.40%) and non-smokers (HA - 89.39%; CHCP - 91.34%). Islam was the dominant religion for most participants. 8.08% HAs and 3.94% CHCPs had a cancer affected family member.

### Assessment of knowledge about cancer

In the knowledge section of the study, the results were mixed (Table 2). In response to items on risk factors or warning signs of cancer, respondents answered correctly for some categories but not for others. Most respondents were not acquainted with advanced cancer treatment options such as - targeted therapy, hormone therapy or immunotherapy. They knew about chemotherapy (HA-86.36% and CHCP-90.55%) but not so much about radiotherapy (HA-54% and CHCP-51.18%). A positive aspect is that most respondents answered correctly about diagnostic tests for cancer. In terms of knowledge about cancers preventable through vaccination, the respondents did not know well about liver cancer (HA-32.83% and CHCP-25.2%).

Only 20.71% HAs and 25.2% CHCPs knew about the availability of all treatment options in Bangladesh and were also unsure about whether such treatments were available at public facilities or not. They knew poorly about govt. screening program for breast, cervical and lung cancers or that these programs were free of cost in govt. hospitals (HA - 51.01%; CHCP - 48.82%). 97.98% HAs and 98.43% CHCPs responded that they did not have enough training to treat cancer. A majority also reported not receiving any training on cancer from the govt.

### Assessment of attitude towards cancer

Table 3 shows that the respondents did not fare well in some items of the attitude section. The most notable deficiency (HA-39.9% and CHCP-40.16%) was seen in response to the question about confidentiality of cancer patients. Few (HA-28.28% and CHCP-33.86%) thought that cancer patients should not think less of themselves due to their condition. A large percentage of respondents (HA-45.45% and CHCP-33.86%) also did not know about the severity of cancer in Bangladesh.

### Assessment of practice regarding cancer

The respondents did well in the practice section of our survey (Table 4). The lowest percentage (HA-74.24% and CHCP-80.31%) of correct answers was in response to the question - “If referred, does a patient actually go to the Upazilla Health Complex?”. Importantly, most respondents (HA-99% and CHCP-95.28%) did not advise Ayurvedik or Homeopathic treatment to cancer patients. Most HAs (93.70%) and CHCPs (93.94%) educated people about cancer during their field visits.

### Overall scoring

Table 5 shows that the knowledge section had a total score of 74 and the mean score was 46.77 (SD - 6.57). We found that 54.15% of the respondents had good or above average score in the knowledge section. Out of 15, the mean score was 9.67 (SD 2.18) and 58.15% CHCPs and HAs had above average score in the attitude section. The respondents had a mean of 5.43 (SD 0.93) out of 6 in the practice section and 65.54% of respondents scored above the average score.

Multivariate analysis in Table 6 also shows that being married (OR 3.3), having cancer in the family (OR 2.48) and working in specific locations led to significantly higher odds of obtaining a good knowledge score. CHWs from Moulvibazar, Sylhet were almost 6 times (OR 5.72) and Barisal, Barisal were 4 times (OR 4.50) more likely to score above average than respondents from Gopalganj, Dhaka. Respondents with a longer duration of employment had lower odds of getting a good score (11–20 years - OR 0.94; > 20 years – OR 0.91) than those who were employed for a shorter duration (< 10 years). We observe that female CHCPs and HAs have slightly higher odds (OR 1.15) of scoring above average than their male counterparts. Also, high income and smoking resulted in lower odds of scoring well in the knowledge section.

From multivariate analysis (Table 6) we observe that, compared to less than 10 years of employment, those employed for 11–20 years had a much lower odds (OR 0.40) and those employed for more than 20 years have a much higher odds of scoring above average (OR 2.34) attitude scores. Higher monthly income (> 25,000 bdt) had lower odds of scoring good (OR 0.29) than those with low income (< 15,000 bdt). Our analysis shows that Muslim respondents had higher odds of scoring above average (OR 1.64) than those belonging to other faiths. Respondents from Moulvibazar, Sylhet (OR 3.11) and 51–60-year-olds had higher odds (OR 2.09) of scoring good; while CHCPs had lower odds (OR 0.75) of doing good than HAs. Respondents with a cancer affected family member had higher odds (OR 1.56) of good attitude than those without such experience.

For practice scores, Table 6 shows that those working in Moulvibazar, Sylhet (OR 7.24), Lalmonirhat, Rangpur (OR 3.99) and Natore, Rajshahi (OR 3.53) had higher odds of scoring well than Gopalganj, Dhaka. Respondents with cancer in their family had higher odds (OR 3.80) and higher income (> 25,000 bdt) had lower odds (OR 0.17) of scoring well. CHCPs had higher odds (OR 1.21) of having good practices than HAs. Those aged 31–40 had slightly lower odds (OR 0.80) of scoring well than those aged 21–30. However, the odds increased with the age group of 51–60 (OR 1.10).

## Discussion

The present study explored cancer related KAP among CHCPs and HAs in rural areas of Bangladesh. Despite limitations, this study is the first attempt to systematically document cancer related KAP among the healthcare workforce in rural Bangladesh.

The results provide support for the view that awareness about cancer, its signs and prevention methods is very limited among rural CHWs. This is concerning as CHCPs and HAs are frontline healthcare workers who are responsible for educating people about certain diseases, including cancer. Similar findings were seen in rural Nepal [24] and rural India [25]. This study highlights the need to increase cancer related KAP among CHCPs and HAs.

Our study shows that 93% of CHWs conducted cancer related teaching or counselling activities during their field visits. This is an important feature in rural areas, where CHCPs and HAs frequently provide counselling services to patients. Prior studies found an association between higher knowledge and attitude scores with modestly better prevention practices [26]. Another study suggested that nurses and social workers should provide counselling interventions as they exert a much greater collective impact than doctors alone [23].

In the knowledge category, female workers had higher odds of scoring well than their male counterparts. Due to the prevailing conservative socio-religious norms, female healthcare workers play a unique role in rural Bangladesh. Female patients frequent the rural healthcare facilities at a higher rate than males and they may be more receptive to female staff [27]. A study conducted in Tehran, Iran found evidence for this, highlighting the important role of female healthcare workers in raising awareness about cancer [28].

We find that work location plays an important role in terms of cancer related KAP among CHWs. This is exemplified by variations in KAP score across the country, where some areas lag more than others. To ensure equitable access to care for all rural people, this disparity needs to be addressed [29] and better performing UHCs need to be recognized for their efforts [30].

The multivariate analysis reveals that CHCPs and HAs having family members with cancer had higher odds of scoring well in all three KAP sections. This may happen because cancer is a complex disease which may have lasting effects on direct caregivers [31]. However, this experience can be repurposed to fill the KAP gaps among coworker CHWs. We also see that age and duration of employment plays an important role in KAP levels. This shows the necessity of targeted training programs – each tailored to specific demographic groups of the rural healthcare workforce [32].

Our respondents did not receive much cancer related training from the govt. The respondents did not have good knowledge about cancer related vaccinations. For a resource poor country like Bangladesh, proper utilization of existing govt. services is a must. The success of govt. vaccination programs depends on the knowledge of rural CHCPs and HAs about existing services. Other studies found similar knowledge deficiencies on cancer related vaccinations, prompting targeted educational initiatives [33]. Johnson et al. also found a large degree of unmet need in terms of training Hong Kong’s hospital-based nurses and social workers [23]. This is particularly concerning as literature shows that training programs improve the workforces’ knowledge and practices [34]. Training of rural CHWs have proven to be successful in preventing and controlling several chronic diseases. Previous studies have found that CHWs without formal professional training can be adequately trained to effectively screen and identify complicated chronic diseases like cardiovascular diseases [35]. Several studies reported that some physicians did not practice cancer screening despite having good knowledge and positive attitude [36–38]. Continued medical education for govt. healthcare workforce was therefore recommended by some authors [39].

The participant CHCPs and HAs had mixed scores regarding knowledge on the signs of cancer. Without a good grasp on the signs of cancer, detection and subsequent referral activities might be hampered. However, we found that a high percentage of CHCPs and HAs referred cancer patients to the UHC or any other specialist physician. This is important as only specialist physicians can determine proper treatment plans for cancer patients [40]. Our results show that CHCPs and HAs are aware of not having the nececssary skills to treat a complex disease like cancer. We also found that CHWs are not aware of the availability of treatment options within Bangladesh or at govt. hospitals. This is important in the referral process as poor knowledge on treatment availability may lead to incorrect or delayed referral to the secondary or tertiary govt. facilities, leading to poor health outcomes [15]. This again highlights the importance of the referral system.

A study conducted in Lazio; Italy found that many physicians did not follow-up with patients who tested positive for cancer [41]. This lies in contrast with our finding that most CHCPs (85.35%) and HAs (84.25%) follow up on a cancer patient once identified during fieldwork. We see that CHCPs and HAs are performing their specified roles with good practices. They also possess good insight of the needs of their community, which conforms to past findings [41]. Any national policy pertaining to cancer should thus take their input into serious consideration.

### Limitations of the study

We acknowledge the caveats of systematic response bias such as socially desirable responses in the attitude and practice sections. Although our large sample size and relatively high response rate reassures the validity of the results. Our study employed convenience sampling in the second stage of the sampling process and thus the sample might not be representative of all CHCPs and HAs. The study was conducted at rural areas of Bangladesh and thus the study results are not representative of urban sections of the country. Due to time and resource constraints, our study employed quantitative methods. Previous studies have found that qualitative methods may also provide important insights to develop an appropriate cancer training module for the workforces. However, this study serves as a baseline for cancer related KAP among the rural healthcare workforces.

## Conclusions

Our findings show that CHCPs and HAs have a significant gap in the category of Knowledge. Although the respondents are working at the lowest tier of the healthcare system of Bangladesh and are not expected to have extensive knowledge on clinical aspects of cancer care, the low scores are still concerning. Similar gaps in attitude and practice were also found. Our study makes the case for improving cancer related KAP of CHWs working in rural parts of Bangladesh. This is essential as part of govt. efforts to control non-communicable diseases. Finally, more research is needed to fully understand the issues relevant to cancer related KAP among other categories of the healthcare workforce, who are working at different levels of the healthcare system of Bangladesh.

## Supplementary Information

**Additional file 1.** Questionnaire.

## Data Availability

The datasets used and analyzed during the current study are available from the corresponding author on reasonable request.

## Abbreviations

CC: Community Clinic
CHCP: Community Health Care Provider
CHW: Community Health Worker
DGHS: Directorate General of Health Services
HA: Health Assistant
HSC: Higher Secondary Certificate
KAP: Knowledge, attitudes, and practices
UHC: Upazilla Health Complex
UHFPO: Upazilla Health and Family Planning Officer
MDG: Millennium Development Goals
MOHFW: Ministry of Health and Family Welfare
SSC: Secondary School Certificate
