# Supplementary material for: Cancer related knowledge, attitude, and practice among community health care providers and health assistants in rural Bangladesh
Source: BMC Health Serv Res. 2021 Mar 2;21:191. doi: 10.1186/s12913-021-06202-z (PMC7927368; doi:10.1186/s12913-021-06202-z)
Supplement: Supplementary file 1 — Additional file 1. Questionnaire. [file 12913_2021_6202_MOESM1_ESM.docx]

**Cancer Related Knowledge, Attitude, and Practice Among Community Health Care Providers and Health Assistants in Rural Bangladesh**

Questionnaire (English)

Cancer Related Practice

Q1.1 If you realize a patient has Cancer, will you try to treat by yourself?

Yes

No

Q1.2 If you realize a patient has Cancer, do you refer him/her to the Upazilla Health Complex or any other specialist physician?

Yes

No

Q1.3 If referred, does a patient actually go to the Upazilla Health Complex?

Yes

No

Q1.4 If you realize a patient has Cancer, do you advise the patient to take Homepathic or Ayurvedic treatment?

Yes

No

Q1.5 Do you educate people on Cancer during fieldwork?

Yes

No

Q1.6 Do you follow-up on a Cancer patient once identified during fieldwork?

Yes

No

Cancer Related Attitude

Q2.1 Do you feel sympathy towards Cancer affected patients?

Yes

No

Don't know

Q2.2 What type of Cancer patients do you feel more sympathetic to?

Female

Male

I don't differentiate among patients

Q2.3 Are you afraid of Cancer patients?

Yes

No

Don't know

Q2.4 Do you feel hesitant to speak with Cancer patients?

Yes

No

Don't know

Q2.5 Do you think that Cancer patients are socially marginalized?

Yes

No

Don't know

Q2.6 Do you think that Cancer patients are avoided by their friends?

Yes

No

Don't know

Q2.7 Do you think Cancer patients face administrative discrimination in terms of receiving govt. benefits?

Yes

No

Don't know

Q2.8 Do you think a patient is responsible for his own disease/fate?

Yes

No

Don't know

Q2.9 If you realize a patient has Cancer, will you disclose that information to others?

Yes

No

Don't know

Q2.10 If you realize a patient is receiving treatment for Cancer, will you disclose that information to others?

Yes

No

Don't know

Q2.11 Do you think Cancer patients should think less of themselves due to their condition?

Yes

No

Don't know

Q2.12 Do you think Cancer patients should be ashamed of themselves due to their condition?

Yes

No

Don't know

Q2.13 Would you be ashamed of yourself if diagnosed with Cancer?

Yes

No

Don't know

Q2.14 How serious of a disease is Cancer?

Very serious

Moderately serious

Not serious at all

Q2.15 In your opinion, how prevalent is Cancer in Bangladesh?

High

Average

Low

Cancer Related Knowledge

Q3.1 Who can be affected by Cancer?

Female

Male

Anyone can be affected

Q3.2 Is Cancer contagious?

Yes

No

Don't know

Q3.3 Is Cancer a hereditary disease?

All types of Cancer are hereditary

Around half of the types of Cancer are hereditary

A few types of Cancer are hereditary

Don't know

Q3.4 What is/are the risk factor(s) for cancer?

Old age

Tobacco

Alcohol

Physical Inactivity

Physical Activity

Certain infections

Obesity

Unhealthy diet

Chronic kidney disease

Consanguineous marriage

Radiation

Sin

Q3.5 Can Cancer be prevented?

Yes

No

Don't know

Q3.6 (If the answer to 3.5 is yes, then) How can Cancer be prevented?

Screening

Healthy eating

Vaccination

Abstinence from Alcohol and Tobacco

Regular physical exercise

Being Inactive

Abstinence from Consanguineous marriage

Q3.7 What is/are the warning sign(s) of cancer?

Changes in bowel or bladder habit

Numbness or weakness in the limbs

A sore that does not heal

Unusual bleeding or discharge

Frequent miscarriage

Thickening or lump in the breast or any other part of the body

Long lasting cough that produces a white or pink mucus

Indigestion or difficulty swallowing

Difficulty speaking

Intense headache with blurred vision

Weight loss despite of enough eating

Obvious change in a wart or mole

Nagging cough or hoarseness

Swollen legs, feet and ankles

Q3.8 What is/are the treatment option(s) for cancer?

Chemotherapy

Radiotherapy

Physiotherapy

Surgery

Targeted therapy

Hormone therapy

Immunotherapy

Ayurved / Unani

Homeopathy

Spiritual remedy

Others (Please write down the names) ________________________________________________

Q3.9 Can Cancer be completely cured?

Yes

No

Don't know

Q3.10 Can Cancer be prevented through Vaccination?

All types of Cancer are preventable through Vaccination

Some types of Cancer are preventable through Vaccination

None of the types of Cancer are preventable through Vaccination

Don't know

Q3.11 (If the answer to 3.10 is yes, then) What types of Cancer are preventable through Vaccination?

Esophageal Cancer

Blood Cancer

Lung Cancer

Cervical Cancer

Liver Cancer

Q3.12 What are the consequences of incomplete treatment for a Cancer patient?

Patients' status will remain unchanged

Patients' status will improve

Patients' status will deteriorate

Q3.13 Are all treatment options for Cancer available in Bangladesh?

Yes

No

Don't know

Q3.14 What is the diagnostic test for Cancer?

X-ray

Endoscopy

Each type of Cancer has a different diagnostic test

Q3.15 Is there any Cancer Screening Program in govt. hospitals which is free of cost?

Yes

No

Don't know

Q3.16 (If the answer to 3.15 is yes, then) What types of Cancer are screened as part of this program?

Lung Cancer

Breast Cancer

Cervical Cancer

Prostate Cancer

Q3.17 What are the types of Cancer you find most frequently during field work?

Cervical Cancer

Lung Cancer

Breast Cancer

Lymphoma

Others (Please write down the names) ________________________________________________

Q3.18 Do you know about the National Cancer Program?

Yes

No

There is no such govt. program

Q3.19 Do you have enough training to treat Cancer?

Yes

No

Q3.20 Have you received any govt. training on Cancer?

Yes

No

Q3.21 What is your usual source for information on Cancer?

Media

Pharmaceutical Company

Govt. Training

I don't receive information on Cancer from any sources

Others (Please write down the names) ________________________________________________

Q3.22 What is the average life expectancy for Cancer patients?

6 months

2 years

It cannot be clearly determined

Q3.23 Where can you treat Cancer?

Public hospital

Private hospital

Public-Private both

Q3.24 Write down the names of a few govt. hospitals where treatment for Cancer is available?

_______________________________________________________________

Q3.25 Write down the names of a few private hospitals where treatment for Cancer is available?

________________________________________________________________

Demographic Information

Q4.1 What is your age?

________________________________________________________________

Q4.2 What is your sex?

Female

Male

Others

Q4.3 What is your marital status?

Single

Married

Others

Q4.4 What is your religion?

Islam

Hinduism

Buddhism

Christianity

Others

Q4.5 How long have you been employed?

________________________________________________________________

Q4.6 What is your designation?

CHCP

Health Assistant

Q4.7 Where do you work?

Upazilla Health Complex

Union Sub-center

Community Clinic

Q4.8 What is the highest level of your education?

SSC

HSC

Diploma

Honors

Masters

Q4.9 What is your average monthly income?

________________________________________________________________

Q4.10 Do you have any Cancer patients in your family?

Yes

No

Q4.11 Do you smoke or use tobacco products?

Yes

No
